# Preliminary Results of Clinical Experience with Consolidative High-Dose Thoracic Radiotherapy for Patients with Extensive-Stage Small Cell Lung Cancer

**DOI:** 10.3390/tomography11050055

**Published:** 2025-05-07

**Authors:** Hakyoung Kim, Jeongeun Hwang, Sun Myung Kim, Dae Sik Yang

**Affiliations:** 1 Departments of Radiation Oncology, Korea University Guro Hospital, Korea University College of Medicine, Seoul 02841, Republic of Korea; sunmyung01@hanmail.net; 2 Department of Medical IT Engineering, College of Software Convergence, Soonchunhyang University, Asan 31538, Republic of Korea; hwangje02@sch.ac.kr

**Keywords:** small cell lung cancer, extensive stage, radiotherapy, local control, survival

## Abstract

Objectives: Extensive-stage small-cell lung cancer (SCLC) has a poor prognosis, but recently, the combination of immunotherapy and chemotherapy has improved treatment outcomes in some patients, and treatment plans may vary depending on the individual’s general condition and tumor response. In addition, intrathoracic tumor control remains a major challenge for this disease. In the current study, we aim to share our clinical experience and demonstrate that consolidative high-dose thoracic radiotherapy effectively reduces intrathoracic tumor recurrence while maintaining acceptable treatment-related toxicities. Materials and Methods: The medical records of 81 SCLC patients treated at Korea University Guro Hospital from January 2019 to December 2023 were reviewed retrospectively. Among them, 22 patients with extensive-stage SCLC who had a favorable tumor response after systemic therapy, including those with oligo-progressive disease limited to the thoracic region and suitable for curative local therapy, received consolidative radiotherapy. A total dose of 52.5 Gy in 25 fractions was administered over 5 weeks to all patients with extensive-stage SCLC. Results and Conclusions: The median follow-up time was 22 months (range: 8–59 months), with the median follow-up period after completing consolidative radiotherapy being 13 months (range: 4–35 months). In-field local recurrence occurred in only one patient after consolidative thoracic radiotherapy. Most importantly, 10 patients with oligo-progressive disease at the thoracic site, at the time of tumor response, remained stable without further intrathoracic in-field recurrence. Additionally, no severe cases of radiation pneumonitis or esophagitis were observed. Based on our institution’s experience, consolidative high-dose thoracic radiotherapy is well-tolerated and associated with fewer intrathoracic recurrences, leading to improved long-term survival in carefully selected patients with extensive-stage SCLC. Given these findings, we believe consolidative radiotherapy should be considered more proactively in clinical practice. Furthermore, these results may help guide the design of future clinical trials.

## 1. Introduction

Recently, systemic regimens for extensive-stage small-cell lung cancer (SCLC) have included programmed death ligand 1-targeted immune checkpoint inhibitors, such as atezolizumab or durvalumab, with etoposide plus platinum [1]. Based on the phase 3 randomized trials IMpower133 [2,3] and CASPIAN [4,5], immunotherapy during and after systemic therapy has become a standard approach for extensive-stage SCLC. Remarkable advances in systemic therapy, such as targeted and immuno-oncologic agents, have aroused great interest in aggressive local therapy for various types of cancers [6,7,8,9,10,11,12,13,14]. In particular, local radiotherapy has been proven to improve local control rates and survival in patients with limited metastatic non-small cell lung cancer (NSCLC) [15,16,17,18,19,20,21]. Recently, consolidative radiotherapy has been shown to have a potential role in extensive-stage SCLC, especially in patients who have shown a favorable response to prior systemic therapy. A randomized trial by Jeremic et al. [22] of consolidative radiotherapy (54 Gy in 36 fractions) and CREST, a Dutch phase 3 randomized trial [23] of palliative low-dose radiotherapy (30 Gy in 10 fractions), have demonstrated that consolidative radiotherapy is well tolerated, results in fewer local recurrences, and improves long-term survival. The NCCN guidelines [1] recommend consolidative thoracic radiotherapy in selected patients with extensive-stage SCLC who respond to first-line chemotherapy and have limited extra-thoracic tumor burden.

Extensive-stage SCLC has a poor prognosis, but recently, the combination of immunotherapy and chemotherapy has improved treatment outcomes in some patients, and treatment plans may vary depending on the individual’s general condition and tumor response. In addition, intrathoracic tumor control remains a major challenge for this disease. The majority of patients had persistent intrathoracic disease after initial systemic therapy and experienced intrathoracic disease progression within the first year of diagnosis. Against this backdrop, the authors believe that more aggressive intrathoracic local therapy is necessary. In the current study, the authors aim to share their clinical experience and demonstrate that consolidative high-dose thoracic radiotherapy effectively reduces intrathoracic tumor recurrence while maintaining acceptable treatment-related toxicities.

## 2. Materials and Methods

### 2.1. Patients

The study was approved by the institutional review board (no. 2022GR0196). The medical records of 81 patients with SCLC treated at Korea University Guro Hospital from January 2019 to December 2023 were retrospectively reviewed. Among them, the 59 patients who had limited-stage SCLC were treated with definitive concurrent chemoradiotherapy. The remaining 22 patients who had extensive-stage SCLC and showed favorable tumor response after systemic therapy, including oligo-progressive disease at the thoracic site only which was amenable to curative local therapy, were treated with consolidative radiotherapy.

### 2.2. Treatment Scheme

In extensive-stage SCLC, four cycles of systemic therapy with a carboplatin (day 1) plus etoposide (days 1–3) regimen were repeated every 21 days. Recently, an atezolizumab (day 1) regimen was repeated every 21 days as a part of primary and maintenance therapies. Most of the patients (20/22 patients, 90.9%) enrolled in this study were treated concurrently; the remaining two patients were treated before mid-2019.

In the current study, the planned total dose and fractions were uniformly applied according to the protocol. A total dose of 52.5 Gy in 25 fractions was administered over 5 weeks to all patients with extensive-stage SCLC. All patients underwent intensity-modulated radiotherapy (IMRT) for treatment planning. For target delineation, the gross tumor volume (GTV) was outlined using the lung or mediastinal window setting. The internal target volume (ITV) was defined based on four-dimensional CT, considering the patient’s respiratory motion. The clinical target volume (CTV) was created by expanding the GTV-ITV by 5 mm in all directions, with adjustments made to accommodate nearby normal anatomical structures. The planning target volume (PTV) was then established by further expanding the CTV by 5 mm. The prescription guideline required that at least 97% of the prescribed dose be delivered to 95% of the PTV. The minimum and maximum doses to 1cc of the PTV were 95% and 107%, respectively. The lung volume receiving ≥20 Gy was limited to ≤35%, while the mean lung dose was kept at ≤20 Gy. The maximum allowable doses to the spinal cord and esophagus were 45 Gy and 60 Gy, respectively, ensuring compliance with normal organ dose-volume constraints.

### 2.3. Surveillance

Disease progression was assessed during follow-up by contrast-assisted chest/abdomen/pelvic computed tomography (CT) scans every two cycles of systemic treatment and at the end of treatment. Brain magnetic resonance imaging (MRI) with contrast enhancement was repeated after two cycles of systemic therapy in patients with extensive-stage SCLC or brain metastases.

In the evaluation of tumor response, the revised Response Evaluation Criteria in the Solid Tumors guidelines (version 1.1) were used. Common Terminology Criteria for Adverse Events (version 4.03) was referred to in evaluating treatment-related complications.

### 2.4. Statistical Analyses

Locoregional failure was defined as tumor recurrence in the lung or regional lymph nodes following consolidative radiotherapy, while recurrences at other sites were considered distant failure. Disease-free survival (DFS) time was defined as follows: the time between the start date of systemic therapy and the date of the first documentation of disease progression or the latest documented follow-up date. Overall survival (OS) time was defined as follows: the time between the start date of systemic therapy and the date of death from any cause or the latest documented follow-up date. The Kaplan–Meier method was applied to calculate the 2-year OS and DFS rates. IBM SPSS Statistics for Windows (version 24.0; IBM Corporation, Armonk, NY, USA) was used to conduct statistical analyses.

## 3. Results

### 3.1. Baseline Characteristics

Table 1 summarizes the clinical characteristics of the patients. The median age of the study population was 66 years (range: 51–79 years). The patients were mostly male (81.8%) and current or ex-smokers (90.9%). Among 22 patients, 12 patients (54.5%) had one metastatic site at the time of diagnosis, and the remaining 10 patients (45.5%) had two or three metastatic sites. Most of the patients (20/22 patients, 90.9%) were treated with concurrent immuno-oncologic agents. Regarding the aspect of tumor response to systemic therapy, 12 patients (54.5%) showed a partial response or stable disease, and the other (10 patients, 45.5%) showed oligo-progressive disease at the thoracic site only on tumor response evaluation.

### 3.2. Patterns of Failure

The median follow-up time was 22 months (range: 8–59 months), with the median follow-up period after completing consolidative radiotherapy being 13 months (range: 4–35 months).

**Figure 1:**
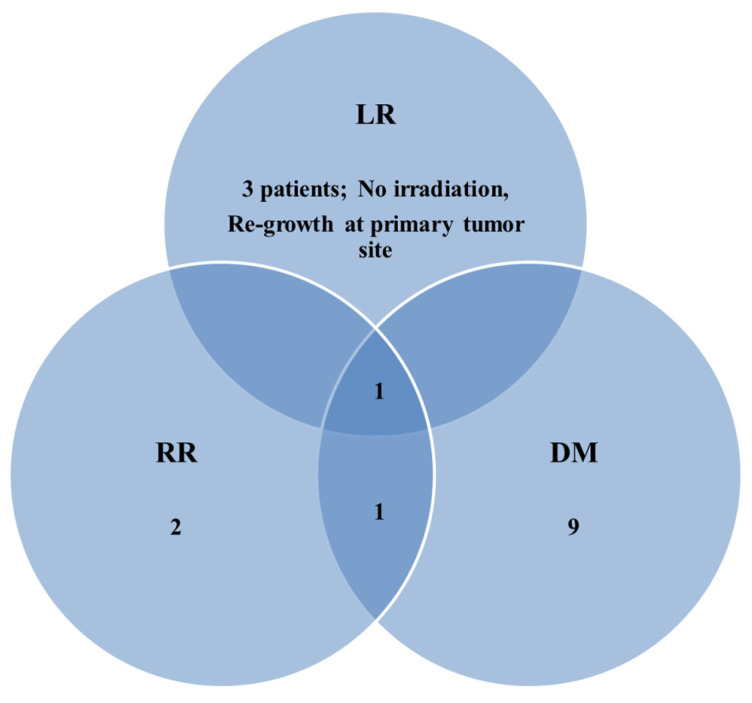
Patterns of failure in patients with extensive-stage small cell lung cancer treated with consolidative radiotherapy. LR, local recurrence; RR, regional recurrence; DM, distant metastasis.

The patterns of failure are shown in Figure 1. Local and regional recurrence after consolidative radiotherapy occurred in four patients, respectively. Interestingly, only one patient showed in-field local recurrence, while the others exhibited re-growth of the primary lung lesion or newly developed regional lymph node recurrence patterns outside the radiation treatment field. Distant metastasis was the most common type of recurrence, occurring in 50.0% of patients (11/22). The most frequent site of distant metastasis was the lung (6/11, 54.5%), followed by the brain (3/11, 27.3%).

With regard to radiotherapy, in-field local recurrence was observed in only one patient. This patient initially demonstrated a favorable tumor response to systemic therapy, and consolidative radiotherapy was administered to the residual primary lung lesion. However, loco-regional recurrence occurred alongside distant metastasis. Ultimately, the patient experienced rapid disease progression and passed away 13 months after completing consolidative radiotherapy. Most importantly, 10 patients with oligo-progressive disease at the thoracic site remained stable without further intrathoracic in-field recurrence.

### 3.3. Treatment-Related Complications and Survival Outcomes

Regarding treatment-related pulmonary toxicity, grade 2 radiation pneumonitis requiring outpatient steroid treatment occurred in 22.7% of patients (5/22). Notably, no cases of severe radiation pneumonitis (grade 3 or higher) were observed in this study. As for other treatment-related toxicities, grade 2 radiation esophagitis requiring medication was reported in three patients (3/22, 13.6%). No severe complications associated with consolidative radiotherapy were observed in any of the patients.

**Figure 2:**
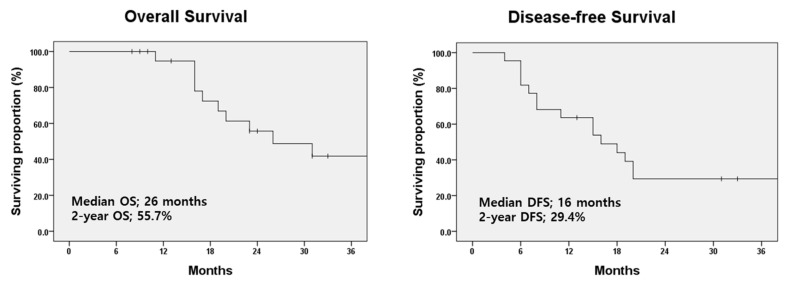
Survival curves in patients with extensive-stage small cell lung cancer treated with consolidative radiotherapy. OS, overall survival; DFS, disease-free survival.

**Figure 3:**
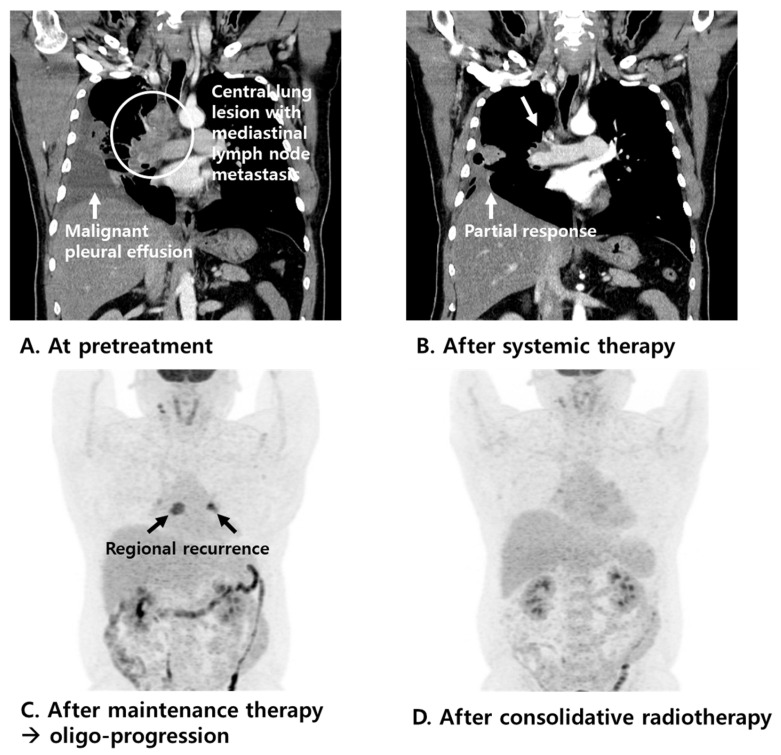
Chest CT images (**A**) at pretreatment and (**B**) after systemic therapy (atezolizumab plus etoposide plus carboplatin) in a patient with extensive-stage SCLC who showed partial response on tumor response evaluation. PET-CT images (**C**) after atezolizumab maintenance therapy and (**D**) after consolidative radiotherapy.

The median OS and DFS were 26 months and 16 months, respectively. The 2-year OS and DFS rates for patients who received consolidative radiotherapy were 55.7% and 29.4%, respectively (Figure 2). The clinical courses of the 10 patients with extensive-stage SCLC who showed oligo-progressive disease at the thoracic site on tumor response evaluation after systemic therapy and were treated with consolidative radiotherapy are shown in Table 2. All patients were treated with immuno-oncologic agents (atezolizumab plus etoposide plus carboplatin) and most of the patients showed well-controlled disease status. One of those patients, who had pleural metastasis at the initial diagnosis, was treated with systemic therapy (atezolizumab plus etoposide plus carboplatin). Three months later, the patient showed a partial response on the tumor response evaluation, and the patient received atezolizumab maintenance therapy (Figure 3). After 10 months, the chest CT during the treatment response evaluation showed regional recurrence, and positron emission tomography-CT (PET-CT) was performed. PET-CT images showed high 18F-fluorodeoxyglucose uptake in the lymph nodes in the left interlobar and subcarinal areas, and no other distant metastases were found. After a multidisciplinary discussion, consolidative IMRT was conducted for the metastatic lymph node lesions with a total dose of 52.5 Gy in 25 fractions. After 10 months, PET-CT imaging evaluating the disease state showed a markedly decreased intensity of metastatic lesions, with no other distant metastases. The patient is alive without further disease progression.

## 4. Discussion

Based on the phase 3 randomized trial IMpower133 [2,3], the NCCN guidelines recommend carboplatin plus etoposide plus atezolizumab as the standard first-line systemic therapy regimen, followed by atezolizumab for maintenance, for patients with extensive-stage SCLC. Conversely, based on the phase 3 randomized trial CASPIAN [4,5], the NCCN guidelines recommend durvalumab plus etoposide plus carboplatin or cisplatin as the standard first-line systemic therapy regimen, followed by durvalumab for maintenance, for patients with extensive-stage SCLC. However, these studies did not include consolidative radiotherapy.

Recently, consolidative radiotherapy has been shown to have a potential role in extensive-stage SCLC, especially in patients who have shown a favorable response to prior systemic therapy [24,25,26]. A randomized trial by Jeremic et al. [22] assessed consolidative thoracic radiotherapy in patients who showed a complete response at distant metastatic sites and at least a partial response at the primary site after three cycles of cisplatin plus etoposide chemotherapy. Patients were randomized to either thoracic radiotherapy (total dose of 54 Gy in 36 twice-daily fractions) in combination with chemotherapy followed by two additional cycles of chemotherapy or four cycles of chemotherapy alone. The thoracic radiotherapy with chemotherapy group showed better local control and survival than the chemotherapy alone group, with a median survival of 17 vs. 11 months (*p* = 0.041). With respect to treatment-related toxicity, pulmonary toxicity did not significantly differ between groups (5% with grade 3 toxicity), whereas the thoracic radiotherapy with chemotherapy group showed higher rates of grade 3 or greater esophageal toxicity (27% of the patients) than the chemotherapy alone group.

The Dutch phase 3 randomized trial CREST [23] also showed a significant survival benefit in patients with extensive-stage SCLC with any response following standard systemic chemotherapy. Patients were randomized to either the PCI and thoracic radiotherapy group or PCI alone group. Interestingly, palliative low-dose radiotherapy with a total dose of 30 Gy in 10 fractions showed improved 2-year OS (13% in the thoracic radiotherapy arm vs. 3% in the control group, *p* = 0.004), whereas the primary outcome of 1-year OS was not significantly different between the two groups (33% vs. 28%, *p* = 0.066). Additionally, no severe toxicity was seen with the addition of thoracic radiotherapy. Furthermore, CREST showed that, compared with patients with three or more metastatic sites, those with two or fewer sites had improved survival and a significant benefit from thoracic radiotherapy. This means that metastatic disease burden is an important prognostic factor and may help select patients who are most likely to benefit from thoracic radiotherapy.

In this study, we defined limited metastatic disease as the presence of one to three metastatic sites at the time of diagnosis. We aimed to include extensive-stage SCLC patients with a limited metastatic burden who demonstrated a favorable tumor response to initial systemic therapy, including those with oligo-progressive disease confined to the thoracic site and suitable for curative high-dose local treatment. The majority of patients (20 out of 22; 90.9%) received concurrent atezolizumab as part of both primary and maintenance therapy. Consequently, the clinical outcomes observed were more favorable than previously reported data. Specifically, we found that the median OS and DFS were 26 months and 16 months, respectively. Additionally, the 2-year OS and DFS rates for patients treated with consolidative radiotherapy were 55.7% and 29.4%, respectively. Additionally, regarding side effects, our institution provides thorough education on recognizing and managing both acute and chronic complications of radiotherapy, including radiation esophagitis and pneumonitis. To mitigate the risk of radiation esophagitis, we recommend that patients start taking Almagel at the beginning of treatment. They are also advised to avoid hot beverages, spicy foods, and other irritants that may aggravate esophageal discomfort. Upon completing treatment, patients receive guidance on the timing, symptoms, and appropriate management of potential radiation pneumonitis.

The current NCCN guidelines recommend consolidative radiotherapy for patients with low-bulk extra-thoracic disease who respond well to initial systemic therapy. However, several factors still need to be considered. First, in clinical practice, even when the initial response to immune-chemotherapy is favorable, the decision to recommend consolidative radiotherapy varies among hospitals and clinicians. Second, standardized data on the optimal treatment timing and regimen, including the total radiotherapy dose and fractionation schedule, are lacking. The role of consolidative thoracic radiotherapy in extensive-stage SCLC remains highly variable. This study has certain limitations. First, as a retrospective analysis, it is subject to potential selection bias. Second, the small sample size limits the demonstration of clinical significance. Despite these limitations, we aim to share our clinical experience and demonstrate that consolidative high-dose thoracic radiotherapy effectively reduces intrathoracic tumor recurrence while maintaining acceptable treatment-related toxicities. Our findings showed that in-field local recurrence occurred in only one patient after consolidative thoracic radiotherapy. Most importantly, 10 patients with oligo-progressive disease at the thoracic site, at the time of tumor response, remained stable without further intrathoracic in-field recurrence. Additionally, no severe cases of radiation pneumonitis or esophagitis were observed, and all patients managed these side effects well with outpatient-prescribed medications.

## 5. Conclusions

Based on our institution’s experience, consolidative thoracic radiotherapy is well-tolerated and associated with fewer intrathoracic recurrences, leading to improved long-term survival in carefully selected patients with extensive-stage SCLC. Given these findings, we believe consolidative radiotherapy should be considered more proactively in clinical practice. Furthermore, these results may help guide the design of future clinical trials.

## Figures and Tables

**Table 1:** Clinical characteristics of patients treated with consolidative radiotherapy (N = 22).

Characteristics	Number	%
Age [years; median (range)]	66 (51–79)	
Sex		
Female	4	18.2%
Male	18	81.8%
Smoking Status		
Never smoker	2	9.1%
Current or Ex-smoker	20	90.9%
ECOG performance status		
0–1	22	100%
2	0	0%
Underlying pulmonary disease		
None	14	63.6%
COPD	7	31.8%
IPF	1	4.6%
Number of metastatic sites at diagnosis		
Single	12	54.5%
Multiple	10	45.5%
M Stage_8th Edition, AJCC		
M1a	9	40.9%
M1b	3	13.6%
M1c	10	45.5%
Immuno-oncologic agent treatment		
No	2	9.1%
Yes	20	90.9%
Response to prior systemic treatment		
Partial response/Stable disease	12	54.5%
Oligo-progressive disease	10	45.5%

ECOG, Eastern Cooperative Oncology Group; COPD, Chronic Obstructive Pulmonary Disease; IPF, Idiopathic Pulmonary Fibrosis; AJCC, American Joint Committee on Cancer.

**Table 2:** Clinical courses of patients who showed oligo-progressive disease on tumor response evaluation after systemic therapy (N = 10).

No.	Age/Sex	Smoking	Underlying Disease	Metastatic Site(s)	Chemotherapy Regimen	Time to Radiotherapy (Months)	Radiotherapy Site(s)	Current Status
1	67/M	Ex-	COPD	Pleura, Bone	Atezo + EP	5	Lung, MLN	LRR (SCN), AWD
2	72/M	Current-	None	Lung, Liver	Atezo + EP	8	MLN, SCN	DM (lung), DOD
3	51/M	Ex-	COPD	Pleura	Atezo + EP	14	MLN	AWD
4	62/M	Ex-	COPD	Brain, Adrenal gland	Atezo + EP	28	Lung, MLN	AWD
5	75/M	Current-	None	Pleura	Atezo + EP	15	Lung, MLN	DM (lung), DOD
6	65/F	Current-	COPD	Lung, lymph node	Atezo + EP	10	Lung	DM (lung), DOD
7	56/M	Ex-	None	Pleura	Atezo + EP	6	MLN, SCN	LRR (primary lung), AWD
8	55/F	Never-	None	Brain	Atezo + EP	8	Lung	RR (MLN), DOD
9	65/M	Ex-	COPD	Pleura, Bone	Atezo + EP	10	MLN	DM (lung), DOD
10	69/M	Ex-	None	Brain, lymph node	Atezo + EP	5	MLN	DM (spinal cord), AWD

COPD, chronic obstructive pulmonary disease; Atezo + EP, atezolizumab plus etoposide plus carboplatin; SCN, supraclavicular lymph node; MLN, mediastinal lymph node; LRR, loco-regional recurrence; DM, distant metastasis; AWD, alive with disease; DOD, dead of disease. All patients were treated with a total dose of 52.5 Gy in 25 fractions.

## Data Availability

The datasets used and/or analyzed in the current study can be obtained from the corresponding author upon reasonable request.
